# Design and development of an aptamer targeting C-type lectin-like molecule-1 as a biomarker for acute myeloid leukemia: a SELEX approach

**DOI:** 10.3389/fbioe.2025.1601453

**Published:** 2025-08-01

**Authors:** Yiwen Chen, Qinhang Li, Shifeng Lou, Hanqing Zeng, Shu Chen

**Affiliations:** Department of Hematology, The Second Affiliated Hospital of Chongqing Medical University, Chongqing, China

**Keywords:** aptamer, C-type lectin-like molecule-1, acute myeloid leukemia, SELEX, molecule

## Abstract

Acute myeloid leukemia (AML), a hematologic malignancy, is an important public health issue. It is a result of the abnormal proliferation of immature myeloid cells. Despite advancements in diagnostic procedures, the early identification of AML remains a significant clinical challenge, marking a distinctive niche for newer theranostic approaches to ameliorate diagnosis and treatment. Aptamers are single-stranded oligonucleotides capable of specific binding with high target affinity that have emerged as a promising candidate for molecular recognition in diagnostics and targeted therapy. In this study, we aimed to select and characterize a high-affinity aptamer for C-type lectin-like molecule-1 (CLL-1), an important cell surface marker for AML. CLL-1-specific aptamers were enriched in the context of iterative positive and negative rounds of selection in a systematic evolution of ligands by exponential enrichment (SELEX) approach. In the following, flow cytometry assessment demonstrated the progression of enrichment and then confirmed their performance. The high-throughput sequencing supported the enrichment of five candidate aptamers. In addition, flow cytometry and specificity assays determined that aptamer-2 specifically bound to CLL-1 with an exceedingly high degree of specificity (94.3%) compared with negative controls and other aptamers. The surface plasmon resonance (SPR) valuation revealed that aptamer-2 has a K_d_ of 1.55 × 10^−8^ M, which indicates a high affinity of binding to CLL-1. Docking analysis reveals a stable and specific interaction between aptamer-2 and CLL-1, highlighting key binding regions and molecular contacts that may underpin targeted recognition. Taken together, the results put forward aptamer-2 as a highly specific and high-affinity candidate for targeting CLL-1. This study opens the prospect of using this aptamer for diagnostic approaches for AML. Further *in vivo* and translational studies on its efficacy and efficiency are needed to elucidate its performance in real-world scenarios.

## 1 Introduction

Acute myeloid leukemia (AML) is a hematologic malignancy characterized by the clonal expansion of immature myeloid cells (Khwaja et al., 2016). According to statistics, in the United States, the age-adjusted incidence of AML is approximately 4.3 per 100,000 individuals annually, with a median age at diagnosis of 68 years (Shallis et al., 2019). AML inflicts a considerable clinical and economic burden, particularly among older patients, and it is well-known as a public health concern (Sacks et al., 2018). Early diagnosis is critical, as timely intervention can improve treatment outcomes and enhance survival rates. However, the lack of sensitive and specific biomarkers often delays diagnosis, highlighting the urgent need for advanced and rapid diagnostic tools to identify AML at its earliest stages and guide the therapeutic strategies (Döhner et al., 2021).

Diagnosing AML presents significant challenges due to the disease’s heterogeneity and the necessity for specialized diagnostic tools, including flow cytometry, cytogenetic, and molecular assessments (Gómez-De León et al., 2023). These complexities can lead to delays in diagnosis, adversely affecting patient outcomes. Recent developments have identified innovative biomarkers, such as C-type lectin-like molecule-1 (CLL-1), a transmembrane glycoprotein from family of C-type lectin-like molecules, predominantly expressed on myeloid cells (on leukemic blasts and leukemic stem cells) (Bakker et al., 2004; Wang et al., 2021). CLL-1 has emerged as a possible target for antibody-mediated immunotherapy in AML and presents more precise and effective diagnosis and treatment options (Shin et al., 2022; Daver et al., 2021; Tashiro et al., 2017).

Aptamers are short, single-stranded DNA or RNA molecules that can selectively bind to specific targets, including proteins, peptides, carbohydrates, small molecules, and even live cells (Röthlisberger and Hollenstein, 2018). They undertake various shapes due to their tendency to form helices and single-stranded loops, enabling high specificity and affinity in binding (Dunn et al., 2017; Zhou et al., 2024).

The identification of aptamers is achieved through the systematic evolution of ligands by the exponential enrichment (SELEX) process. This method starts with a large library of random oligonucleotide sequences (Didarian et al., 2024; Brown et al., 2024). Through repeated cycles of binding, separation, and amplification (positive and negative screening), sequences with the highest affinity for the target are enriched. The SELEX process has been involved in developing aptamers for a wide range of applications, including disease diagnosis and targeted therapies (Zhuo et al., 2017; Zhu et al., 2024).

Aptamers offer several advantages for the early diagnosis of diseases like AML through the detection of biomarkers such as CLL-1. Their ease of chemical synthesis and alteration allows for rapid improvement and optimization of aptamer-based diagnostic tools (Kumar Kulabhusan et al., 2020; Mahmoudian et al., 2024; Domsicova et al., 2024). Moreover, aptamers exhibit high stability across various conditions and low immunogenicity, making them appropriate for clinical applications (Chen et al., 2025).

**FIGURE 1:**
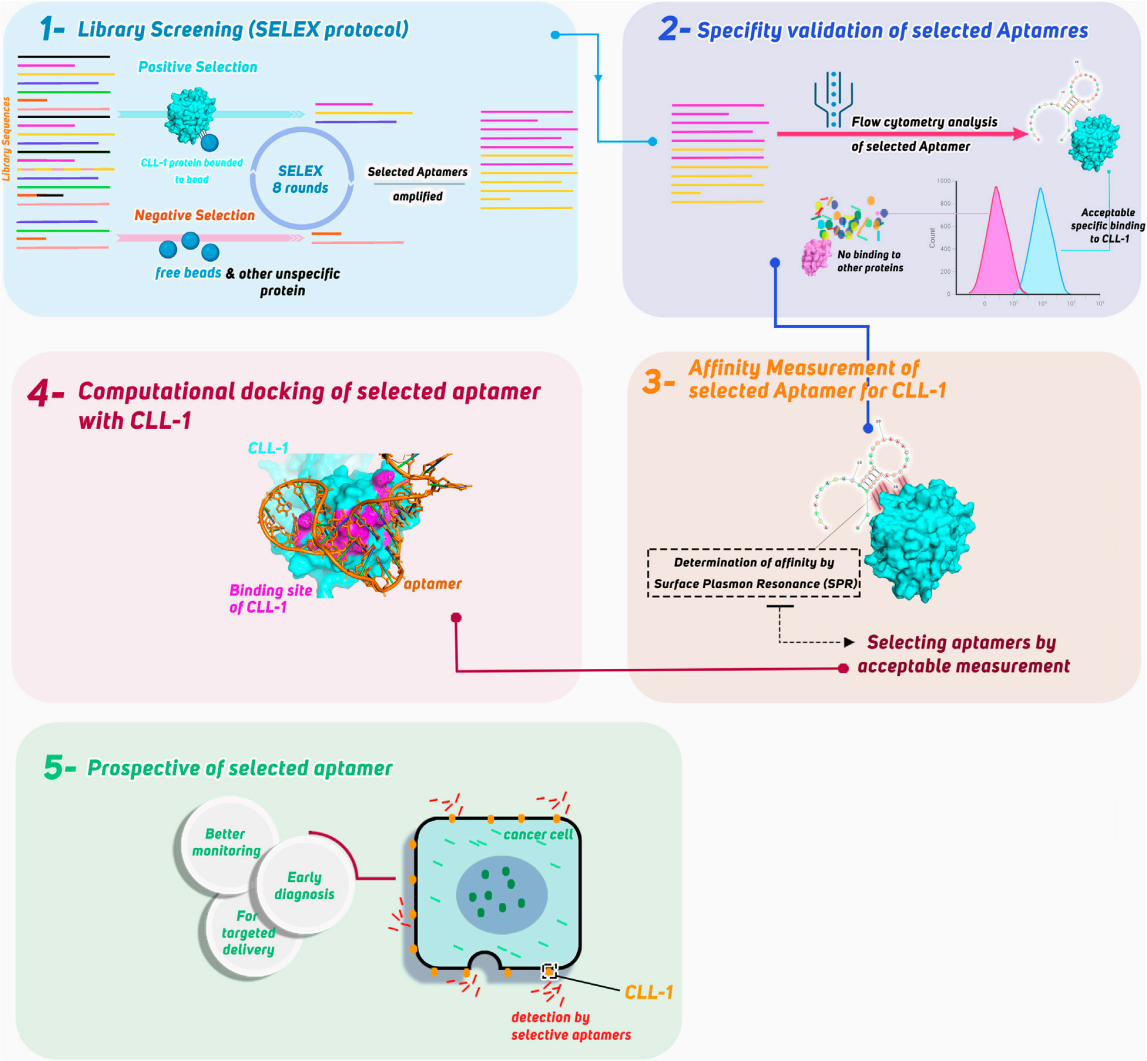
Graphical abstract of this study.

In this study, we aimed to develop a highly specific aptamer targeting the CLL-1 protein for its potential application in AML diagnostics. Utilizing the SELEX protocol, we screened an aptamer library to identify candidates with high affinity for CLL-1. The selected aptamer’s specificity and performance were validated through flow cytometry, where its binding was compared against other controls to ensure accuracy. To further confirm its binding stability, surface plasmon resonance (SPR) was employed to measure the aptamer’s affinity to CLL-1. Finally, bioinformatics simulations were conducted to model and visualize the interaction between CLL-1 and the selected aptamer, providing additional insights into its mechanism of action. In this study, we highlight the potential of the candidate aptamer (aptamer-2) as a robust tool for precise and efficient biomarker detection in AML (Figure 1 – graphical abstract).

## 2 Materials and methods

### 2.1 Library preparation for screening and protein coupling

The first step of SELEX involves preparing the initial ssDNA library and coupling the target protein to magnetic beads using reagents from the Aptomax screening kit (for more details, see Supplementary Material S2). The steps followed are listed below.

#### 2.1.1 Library dissolution and rapid denaturation

• Weigh out the LibP1-76 nt library powder and centrifuge at 12,000 × g for 10 min (room temperature).
• Resuspend the pellet in 280 µL Dulbecco’s PBS (DPBS, pH 7.4) to a final library concentration of 5 µM. Vortex for 15 s, and then centrifuge at 8,000 × g for 30 s to remove any insoluble debris.
• Aliquot 70 µL of this solution into each 0.2-mL PCR tube. Denature the solution by heating at 95°C for 10 min (lid at 105°C), immediately cool on ice for 5 min, then allow to return to room temperature for 5 min before proceeding.

#### 2.1.2 Protein coupling

• Transfer 50 µL of the carboxylated magnetic beads (Amptomax) into a microcentrifuge tube and wash 4× with 200 µL ultrapure water. Resuspend by gentle inversion, magnetize (1 min), and discard the supernatant.
• Prepare the activation mix by combining 50 µL freshly thawed EDC (50 mg/mL in water) with 50 µL NHS (50 mg/mL). Vortex 5 s, and then immediately add to the washed beads. Incubate on an orbital shaker at room temperature (150 rpm) for 20 min, gently inverting every 5 min to prevent aggregation.
• After activation, magnetize the beads, discard the supernatant, and wash once with 200 µL ultrapure water (complete the wash in ≤1 min).
• Meanwhile, prepare 1 mg/mL of the target protein by dissolving recombinant CLL-1 (MedChemExpress) in 40 µL 0.1 M sodium acetate (NaAc) buffer, pH 5.5. (For negative controls, prepare 1 mg/mL bovine serum albumin (BSA) similarly.)
• Add 40 µL of the 1 mg/mL protein solution to the activated beads (bringing the total volume to ∼100 µL with NaAc buffer if needed). Incubate at room temperature on a shaker (150 rpm) for 60 min, gently mixing every 10 min.
• Magnetize the beads, remove any unbound protein, and add 100 µL 1 M ethanolamine (pH 8.5). Incubate on the shaker for 10 min to quench residual NHS esters.
• Finally, wash the beads 4× with 200 µL DPBS (resuspend for 2 s, magnetize for 1 min, and discard). Label the resulting beads as **MB-CLL-1** (or, for controls, **MB-BSA**) and store at 4°C in DPBS until needed.

### 2.2 Secondary library preparation for screening

• Take 70 µL of the denatured library (from Section 1.1) and add it to 50 μL MB-CLL-1 in a total volume of 120 µL DPBS. Incubate at room temperature on an orbital shaker (150 rpm) for 60 min.
• Magnetize for 1 min, discard the supernatant (flow-through), and wash the beads 4× with 200 µL DPBS (vortex for 2 s each wash, magnetize for 1 min, and discard).
• To elute the bound sequences, add 200 µL DPBS and heat the beads at 95°C (water bath) for 10 min. Immediately magnetize, collect the supernatant (“R-Elution”), and place on ice for PCR.

### 2.3 PCR amplification of aptamers

#### 2.3.1 PCR

• Thaw 2 mL PCR Master Mix (Amptomax) at room temperature, centrifuge briefly, and transfer to a 50-mL tube.
• Add up to 40 µL of R-Elution as the template, plus 5 µL forward primer (10 μM, 6-FAM-labeled) and 5 µL reverse primer (10 μM, poly-A spacer). Bring the final volume to 500 µL with nuclease-free water. The primer sequences are listed in Supplementary Table S1.
• Aliquot 62.5 µL into each of eight PCR tubes. Program the Bio-Rad T100 cycler as follows:
  1. 95°C for 3 min
  2. 35 cycles of: 95°C for 30 s → 60°C for 30 s → 72°C for 30 s
  3. Final extension: 72°C for 5 min
  4. Hold at 4°C indefinitely

#### 2.3.2 Concentrating PCR products

• Pool all eight PCRs (∼500 µL) into a 15-mL tube, add 10 mL n-butanol (Sigma), invert 10× to mix, and centrifuge at 3,500 × g for 10 min (room temperature).
• Carefully remove and discard the upper organic and emulsion layers. Collect the lower aqueous phase (∼500 µL) containing dsDNA, transfer to a fresh 1.5-mL tube, and set aside for denaturing PAGE.

### 2.4 Single-strand preparation of aptamers

#### 2.4.1 Sample preparation

• Reserve 5 µL of the concentrated PCR product at −20°C as backup. To the remaining ∼495 μL, add an equal volume of 2× TBE urea loading dye (8 M urea, bromophenol blue; Bio-Technology Co., Ltd., Shanghai), then heat at 95°C for 10 min. Centrifuge briefly (<5 s) and load onto the denaturing gel.

#### 2.4.2 Denaturing PAGE

• Cast an 8% acrylamide:bisacrylamide (19:1) gel in 1× TBE with 8 M urea (0.75 mm thickness). Pre-run at 300 V for 30 min in 1× TBE to equilibrate.
• Load samples and run at a constant 300 V for 1 h. Visualize FAM-labeled bands under a 365- nm UV lamp; excise the 76-nt band corresponding to the library.

#### 2.4.3 DNA recovery

• Place the gel slice into a 0.5-mL crushed gel tube and centrifuge at 12,000 × g for 2 min to pellet fragments. Discard the upper phase and add 1 mL DPBS to the pellet. Boil in a water bath for 10 min, centrifuge at 12,000 × g for 1 min, and collect the supernatant.
• Perform a second wash: add 1 mL fresh DPBS to the pellet, boil 10 min, centrifuge at 12,000 × g for 1 min, and pool supernatants (total ∼2 mL).

#### 2.4.4 Concentration and dialysis

• To the ∼2 mL pooled supernatant, add 11 mL n-butanol (5.5×), invert 10× to mix, and centrifuge at 3,500 × g for 5 min. Discard the upper phases and transfer the lower ssDNA solution (∼200 µL) to a microdialysis device (3.5 kDa MWCO).
• Dialyze against 40 mL DPBS overnight (12–16 h) at 4°C with gentle rocking. After dialysis, collect ∼100–200 µL ssDNA by puncturing the membrane’s bottom, centrifuge at 12,000 × g for 5 min to remove residual debris and store at −20°C as Pool n for the next round.

### 2.5 Repeated screening

• Perform a total of eight SELEX rounds. In Rounds 2–8, use Pool (n − 1) (adjusted to 5 µM in 70 µL) as input and include dual negative selection steps immediately prior to positive binding.

#### 2.5.1 Negative (counter) selection (rounds 2–8)

• Step A: MB Pre-Clearing
  ○ Combine 70 µL Pool (n − 1) with 50 µL unmodified MB in 120 µL DPBS. Incubate at room temperature on a shaker (150 rpm) for 60 min in Round 2, decreasing each round to 30 min by Round 8 (e.g., 60 min → 60 min → 45 min → 45 min → 30 min → 30 min → 30 min → 30 min).
  ○ Magnetize for 1 min and discard the bead pellet (removes sequences that bind beads nonspecifically).
• Step B: MB-BSA Counter-Selection
  ○ Transfer the supernatant from Step A to 50 μL MB-BSA and incubate under the same conditions (time/temperature) as Step A.
  ○ Magnetize for 1 min and discard the bead pellet (removes sequences binding BSA nonspecifically).
• Step C: Washes
  ○ Collect the final supernatant (enriched for CLL-1–specific sequences) and perform 4× 200 µL DPBS washes: for each wash, add DPBS, vortex 2 s to resuspend, magnetize for 1 min, and discard the supernatant. This step mechanically dissociates the weakly bound, off-target sequences.

#### 2.5.2 Positive binding and washes

• Take the washed supernatant from Step 2.5.1 and add to 50 μL MB-CLL-1 (pre-equilibrated in DPBS). Incubate on a shaker (150 rpm) at room temperature for the specified time:
  ○ Round 2: 60 min; Round 3: 45 min; Round 4: 45 min; Round 5: 30 min; Round 6: 30 min; Round 7: 30 min; Round 8: 30 min.
• After incubation, magnetize for 1 min, discard flow-through, then wash beads 4× with 200 µL DPBS for Rounds 2–4, 5× with 200 µL DPBS for Rounds 5–6, or 6× with 200 µL DPBS for Rounds 7–8 (vortex 2 s each, magnetize for 1 min, discard).

#### 2.5.3 Elution and amplification

• Add 200 µL DPBS to the washed MB-CLL-1, heat at 95°C for 10 min (water bath), magnetize for 1 min, and collect supernatant as R-Elution n.
• PCR amplify R-Elution n exactly as in Section 3.1. Reduce the number of PCR cycles by two if non-specific bands appear (Rounds 7–8).

#### 2.5.4 Monitoring by flow cytometry

• After each round, take 1 µg of FAM-labeled pooled ssDNA (from PCR) and incubate with 10 μL MB-CLL-1 in 200 µL DPBS (room temperature, 30 min, 150 rpm). Wash 3× with 200 µL DPBS, resuspend in 200 µL DPBS, and analyze on a Beckman CytoFLEX (FITC channel). Record the percentage of FITC-positive beads. A progressive increase in % FITC positivity indicates successful enrichment; specifically, background binding to MB-BSA drops from ∼10% to 15% in early rounds to <1% by Round 8.

#### 2.5.5 Sequencing checkpoints

• After Rounds 3 and 5, reserve 5 µL of unamplified R-Elution for high-throughput sequencing (Illumina, 36 nt single-end). Analyze read frequencies to identify emerging sequence families.

### 2.6 Aptamer screening and selection

#### 2.6.1 Screening phase and positive/negative selection

• In each round, incubate the ssDNA library with MB-CLL-1 beads at room temperature (150 rpm) for the specified time. Magnetically separate beads to collect bound sequences (positive selection) and discard the unbound sequences. Prior to positive binding, perform dual negative selection (Section 2.5.1) to remove bead- and protein-nonspecific binders.

#### 2.6.2 Sequencing of screening steps

• After PCR amplification of each R-Elution, submit products for Illumina sequencing (36 nt single-end). Analyze read counts to identify enriched sequences and track their frequency over successive rounds.

#### 2.6.3 Aptamer candidates

• From Round 8 sequencing, identify the top five sequences (highest copy numbers). These sequences become candidate aptamers for downstream validation.

### 2.7 Aptamer validation

#### 2.7.1 Testing selected aptamers by flow cytometry

• Synthesize each candidate with a 5′6-FAM label. Incubate 100 nM aptamer with 10 μL MB-CLL-1 in 200 µL DPBS at room temperature (30 min, 150 rpm). Wash beads 3× with 200 µL DPBS, resuspend in 200 µL DPBS, and analyze on the CytoFLEX (FITC channel). Controls: MB-BSA + aptamer, MB-CLL-1 + random library (100 nM), MB-BSA + random library. Report the percentage of FITC-positive beads. Aptamer-2 showed 94.3% binding to MB-CLL-1 vs. 0.26% to MB-BSA.

#### 2.7.2 Aptamer-2 Specificity Validation

• Incubate 100 nM aptamer-2 with 10 μL MB-CLL-1 or 10 μL MB-BSA under identical conditions (30 min, 150 rpm). Wash 3× with 200 µL DPBS, resuspend, and measure binding by flow cytometry (FITC). Specificity is confirmed if > 90% of MB-CLL-1 beads are FITC-positive and <1% of MB-BSA beads fluoresce.

### 2.8 Measurement of aptamer-2 affinity to CLL-1 by surface plasmon resonance (SPR)

The affinity of aptamer-2 to CLL-1 is assessed using SPR. The CLL-1 protein is immobilized on the SPR chip, and aptamer-2 is presented in changing concentrations. The interaction between aptamer-2 and CLL-1 is measured in real time by monitoring changes in the refractive index, allowing for the determination of the binding affinity (K_d_).

### 2.9 Final aptamer candidates

Aptamers with the highest binding affinity and specificity from the flow cytometry and SPR analyses were selected. These aptamers will be considered for further therapeutic or diagnostic applications and need further evaluation and investigation.

### 2.10 Flow cytometry data analysis

The “.fcs” files were loaded and then analyzed with FlowJo v10.10^®^ software.

### 2.11 Secondary structure of selected aptamers

The RNAstructure^®^ webserver (https://rna.urmc.rochester.edu/RNAstructureWeb/Servers/Predict1/Predict1.html) was used to predict the secondary structure of the selected aptamers. The type of nucleic acid was selected as DNA. The default secondary structure prediction options were selected according to the web server instructions. The output of secondary structure with acceptable energy and prediction certainty was designated.

### 2.12 Docking and 3D structure visualization

To explore the interaction between aptamer-2 and CLL-1, the experimentally determined 3D structure of CLL-1 was obtained from the PDB for further analysis (Mori et al., 2024). The 3D structure of aptamer-2 based on its sequence was predicted using Xiao Lab server (http://biophy.hust.edu.cn) by the default recommended settings (Wang et al., 2015; Zhang et al., 2022). The best-scored and most energetically stable 3D structure of aptamer-2 was selected for docking analysis.

We performed molecular docking using LightDock (Jiménez-García et al., 2018) to investigate the interaction between aptamer-2 and CLL-1. The top five ranked and most stable complexes in the docking results were selected for further analysis. We used PyMOL^®^ software to visualize the 3D structures of these docked complexes.

## 3 Results

### 3.1 Aptamer screening and selection

Systematic positive and negative selection rounds are conducted to isolate aptamers with high affinity and specificity for the CLL-1 protein. Positive selection was performed using CLL-1-bound beads to enrich for aptamers capable of binding the target protein, while negative selection was performed with unbound beads to eliminate non-specific binders.

**FIGURE 2:**
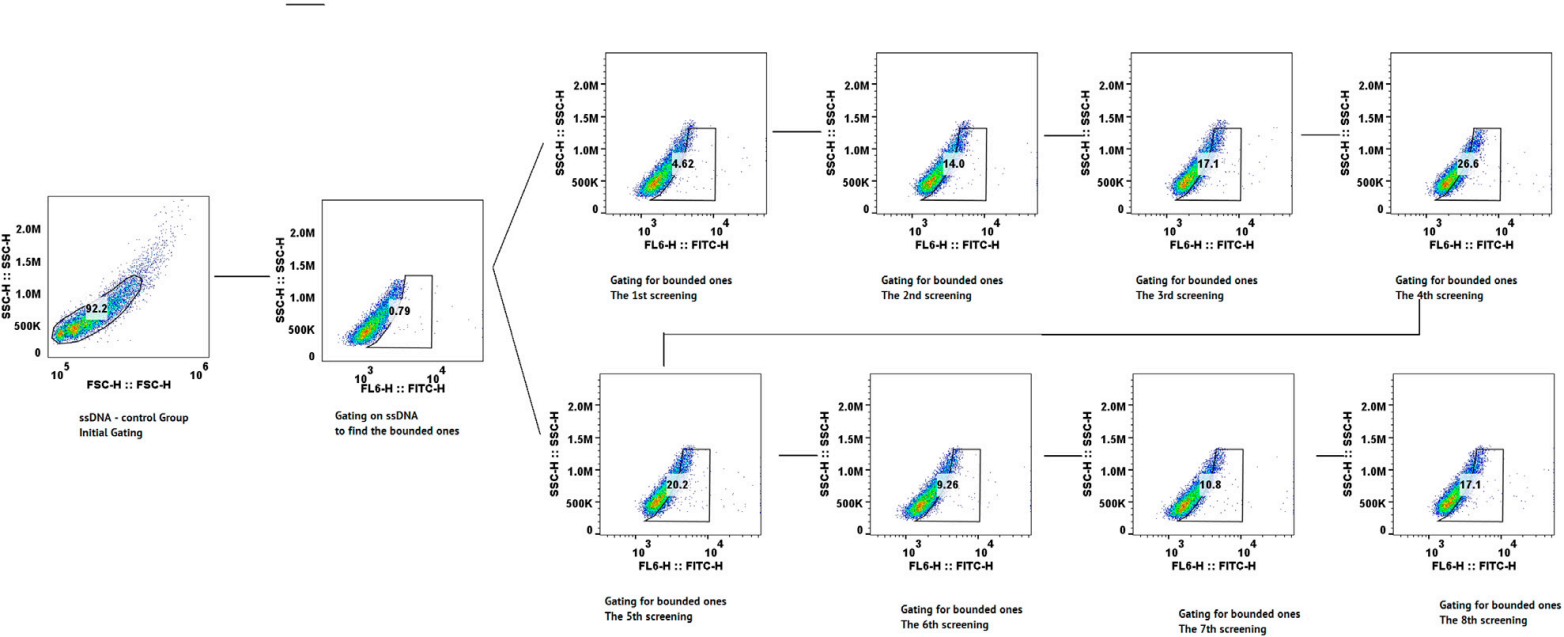
Scatter plot of flow cytometry data from the screening process, showing each cycle step-by-step.

Flow cytometry assessment was implemented after each round to monitor the enrichment of aptamers with binding specificity to CLL-1. As shown in Figure 2, the proportion of the bound aptamers increased progressively across the selection rounds, reflecting the efficiency of the SELEX process in enriching target-specific sequences. Notably, a steady rise in binding was observed through the first few rounds, with a sharp increase seen by the fifth round. However, a marked decrease in binding was detected after round 7, potentially due to the overrepresentation of non-functional sequences or a reduction in the diversity of the aptamer pool, which is more probable. By the eighth round, the binding proportion increased again, indicating the selection of aptamers that were highly specific for CLL-1.

This trend highlights the iterative nature of the SELEX process and the progressive enrichment and specificity of the aptamer candidates at each stage by flow cytometry.

**FIGURE 3:**
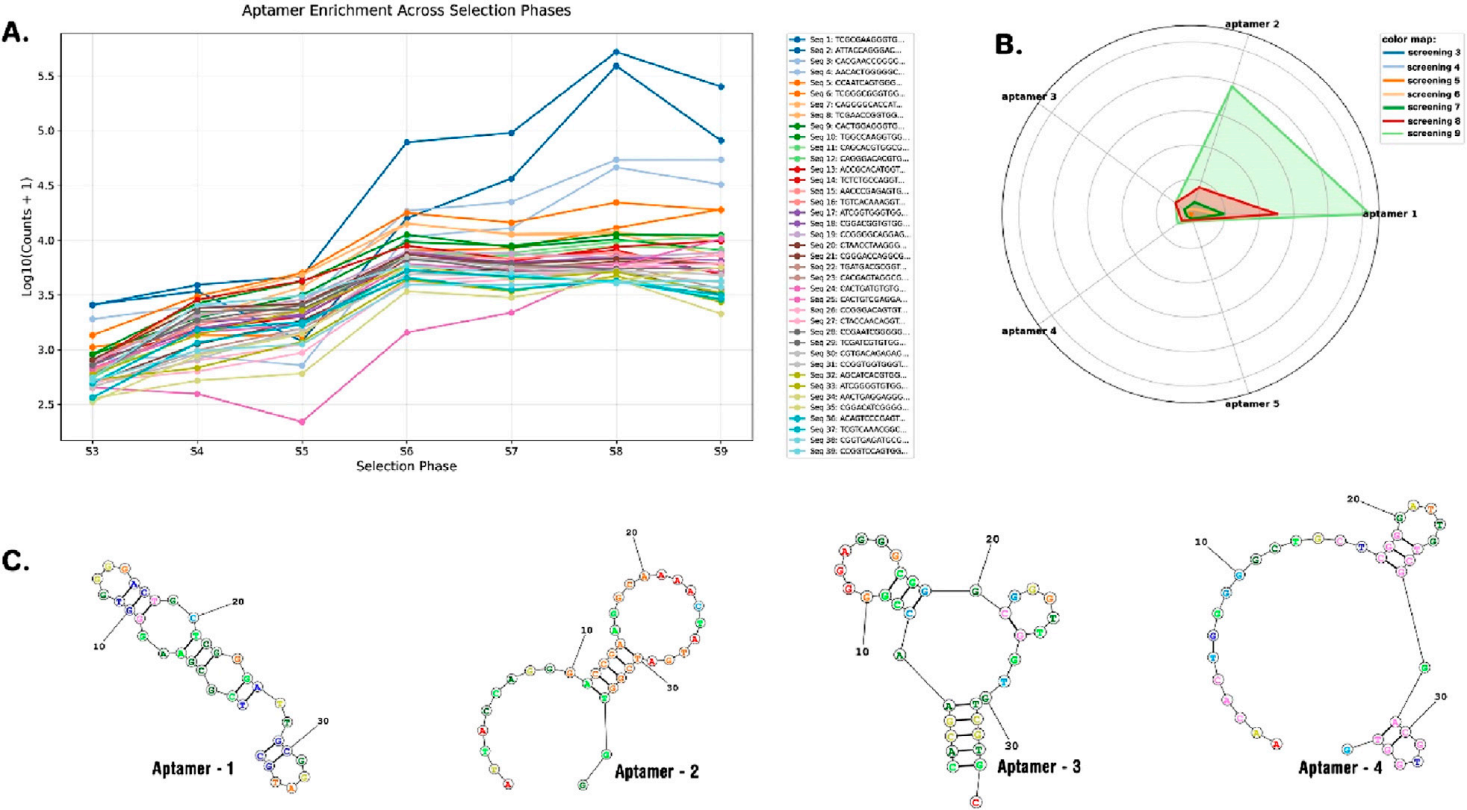
The enrichment of aptamers across different selection phases, listing the copy number of each sequence by each cycle over the log of count **(A)**. Radar plot of aptamer copy number over cycles of screening (aptamer-1, aptamer-2, aptamer-3, aptamer-4, and aptamer-5) **(B)**. 2D structure of selected aptamers **(C)**.

The results of high-throughput sequencing of 36-nucleotide aptamers revealed significant enrichment of five aptamers across seven consecutive cycles of screening (from cycle 3 to cycle 9), as demonstrated in Supplementary Material S1, Figures 2B, 3A; Supplementary Figure S1. Figure 3B illustrates the prominent enrichment of aptamer-1 to aptamer-4 through sequential screening cycles, highlighting their increasing prevalence and potential binding affinity. The sequences of the selected aptamers are detailed in Table 1, while their secondary structures are depicted in Figure 3C. This enrichment pattern underscores the effectiveness of the screening process in identifying aptamers with high specificity and binding potential.

**TABLE 1:** Sequences of selected aptamers and their copy number in sequencing at each screening phase.

Name	Sequence	Total count	S3	S4	S5	S6	S7	S8	S9
Apt-1	TCG​CGA​AGG​GTG​GGG​ACT​GCT​CGG​GAT​TGC​GGA​TGC	957,907	2,556	3,890	4,663	78,200	95,418	521,942	251,238
Apt-2	ATT​ACC​AGG​GAC​CGA​AGG​CAA​AAC​TAT​GAT​CGG​TGG	530,045	2,565	3,406	1,194	15,791	36,425	389,740	80,924
Apt-3	CAC​GAA​CCG​GGG​AGG​GCG​GGC​GGG​TTG​GTG​TCG​TGC	156,134	1,899	2,517	2,542	18,423	22,322	54,162	54,269
Apt-4	AAC​ACT​GGG​GGC​TGC​TCG​GGA​TTG​TCG​GAC​GTG​GTG	104,114	808	876	716	10,657	12,863	46,064	32,130

### 3.2 Aptamer validation

#### 3.2.1 Evaluation of selected aptamers for CLL-1 binding using flow cytometry

**FIGURE 4:**
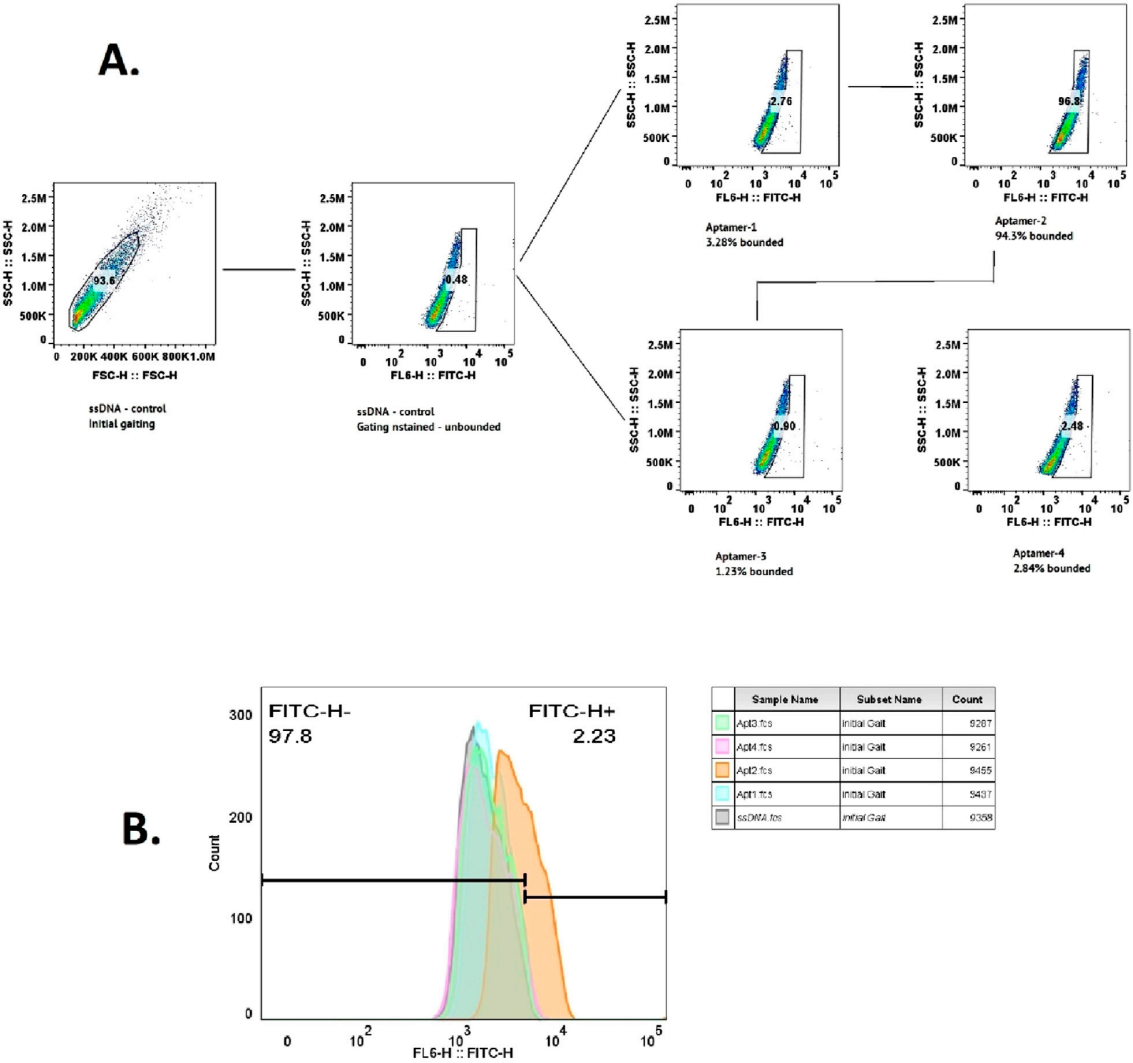
Flow cytometry data for the ssDNA control, showing the initial gating and count measurements. The following flow cytometry data for aptamers 1–4 indicate the percentage of bound aptamers **(A)**. Histogram plot of the same data **(B)**.

To evaluate the binding specificity of the selected aptamers to CLL-1, flow cytometry analysis was conducted for each aptamer individually (Figure 4). The results, illustrated in Figures 3A,B, revealed that aptamer-2 exhibited the highest binding and specificity toward CLL-1 compared to the other candidates. As illustrated in Figure 3A, according to the enrichment pattern across the selection phases, aptamer-2 demonstrated a significant increase in binding during the later rounds, highlighting its superior affinity. Additionally, detailed fluorescence intensity histograms (Figure 4B) demonstrate distinct shifts for aptamer-2, confirming a higher proportion of FITC-positive events, indicative of strong and specific interaction with CLL-1.

This was further validated by comparing the FITC-H+ population, where aptamer-2 accounted for 94.3% of the bound cells, a substantially higher percentage than other candidates, such as aptamer-1 (3.28%) and aptamer-4 (2.84%). These findings collectively demonstrate aptamer-2’s ability to bind significantly to the CLL-1 epitope, positioning it as a highly promising candidate (Figure 3A).

#### 3.2.2 Specificity validation of aptamer-2

**FIGURE 5:**
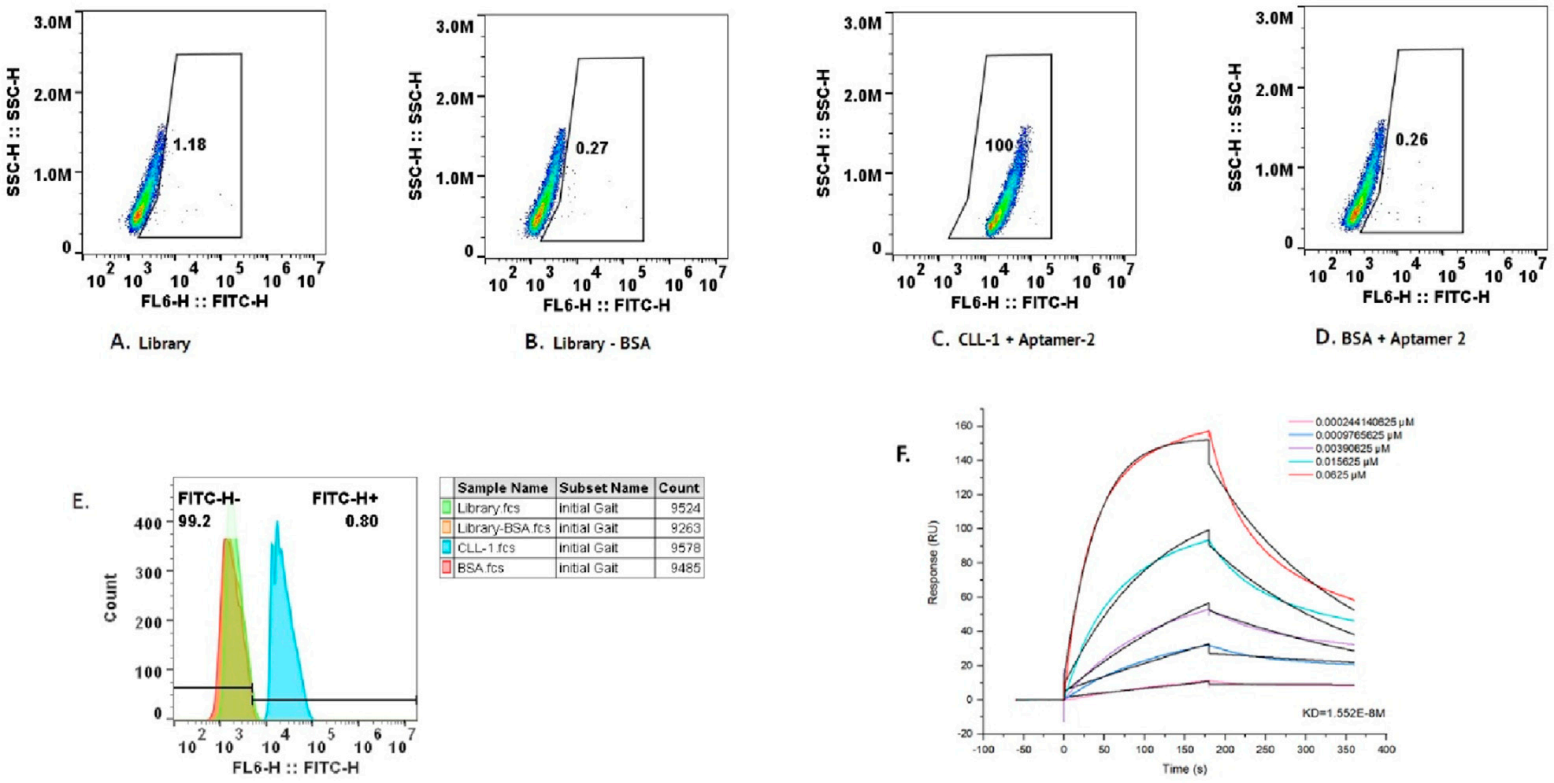
**(A)** Flow cytometry data for BSA with aptamer-2. **(B)** Flow cytometry data for the library. **(C)** Flow cytometry data for the library minus BSA. **(D)** Flow cytometry data for CLL-1 with aptamer-2. **(E)** The cumulative data of the scatter plot in the histogram above. **(F)** Response curve from the binding assay of aptamer-2 and CLL_1, showing the response (RU) over time (s) for different concentrations of the ligand (aptamer-2). It includes a dissociation constant (K_d_) value, indicating the binding affinity of the aptamer.

Flow cytometry analysis was used to further validate the specificity of aptamer-2 for its target, CLL-1, as illustrated in Figure 5. The experiment incorporated multiple controls and conditions, including the aptamer library as a reference, BSA as a negative control to rule out non-specific interactions, and CLL-1 as the target molecule. This study confirmed the absence of non-specific binding of aptamer-2 to the control protein (Figure 5C, BSA + aptamer-2: 0.26%), and on the other hand, high-affinity binding of aptamer-2 to its target, CLL-1 (Figure 5D, CLL-1 + aptamer-2: 100%). It is important to note that the fluorescence profiles of the entire aptamer pool alone and with BSA serve as a baseline for comparison in this part of the study (Figure 5A, Library: 1.18%; Figure 5B, Library: BSA: 0.27%). Finally, the strong fluorescence signal with CLL-1 + aptamer-2 in gating highlights the high-affinity binding of aptamer-2 to its target, CLL-1, with negligible non-specific interactions. These results corroborate the exceptional specificity of aptamer-2, reinforcing its potential for diagnostic and therapeutic applications targeting CLL-1.

### 3.3 Affinity measurement of aptamer-2 for CLL-1 using surface plasmon resonance (SPR)

The affinity of aptamer-2 for its target, CLL-1, was quantitatively evaluated using SPR, a gold-standard technique for measuring molecular interactions with high sensitivity. The SPR sensorgram (Figure 5F) illustrates the binding response (in response units, RU) of aptamer-2 to CLL-1 across a concentration gradient ranging from 0.000244 μM to 0.0625 μM. A clear concentration-dependent increase in binding response was observed, confirming the strong and specific interaction between aptamer-2 and CLL-1.

The equilibrium dissociation constant (K_d_) was determined to be 1.55 × 10^−8^ M, indicating an exceptionally high affinity. This low K_d_ value underscores the ability of aptamer-2 to bind tightly and efficiently to CLL-1, with minimal likelihood of dissociation under physiological conditions. These findings further corroborate the specificity and robustness of aptamer-2, positioning it as an excellent candidate for applications demanding precise molecular recognition, such as diagnostic platforms or targeted therapeutic strategies. The SPR results reinforce its potential utility in advancing biomedical innovations.

### 3.4 Computational docking of aptamer-2 and CLL-1

Aptamer-2 shows promising affinity for CLL-1, but the nature of the interaction remains unclear. To explore this, the experimentally determined 3D structure of CLL-1 was obtained from the PDB for further analysis (Mori et al., 2024). The 3D structure of aptamer-2 based on its sequence was predicted using the Xiao Lab server (http://biophy.hust.edu.cn) with the default recommended settings (Wang et al., 2015; Zhang et al., 2022). The best-scored and most energetically stable 3D structure of aptamer-2 was selected for docking analysis.

**FIGURE 6:**
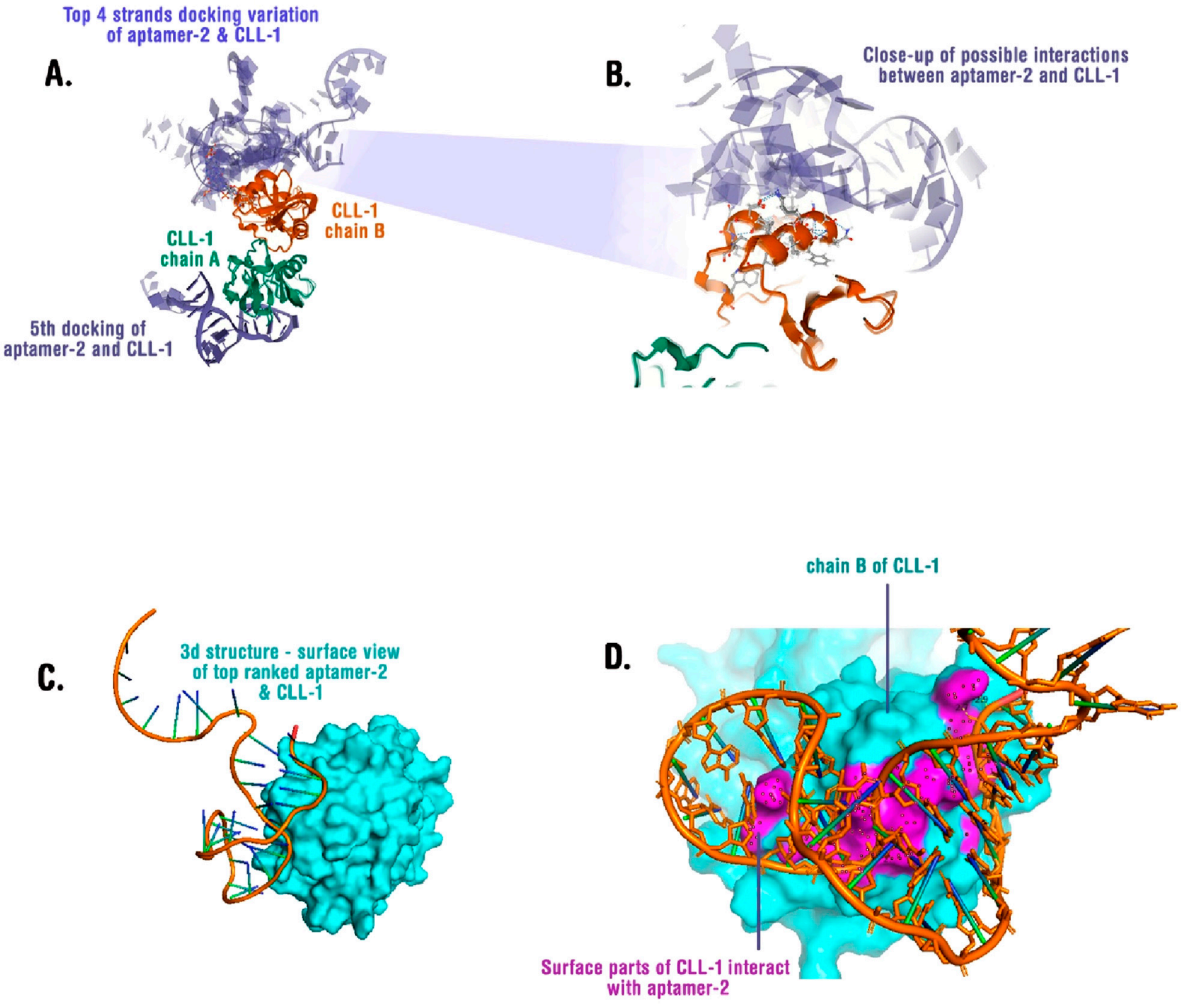
**(A)** Superimposed docking results showing the top four binding poses of aptamer-2 with CLL-1. Chain A (green) and chain B (orange) of CLL-1 are depicted, illustrating potential binding variations. **(B)** A close-up view of the interaction interface between aptamer-2 and CLL-1, highlighting key molecular contacts. **(C)** Surface representation of CLL-1 (cyan) in complex with the top-ranked aptamer-2 binding conformation, revealing the structural fit. **(D)** Interaction map showing the surface regions of CLL-1 (cyan) that engage with aptamer-2 (orange), with interacting residues highlighted in magenta.

To investigate the interaction between aptamer-2 and CLL-1, we performed molecular docking using LightDock (Jiménez-García et al., 2018). This process helps identify the most favorable interaction conformations. The top five docking results revealed that Aptamer-2 predominantly binds to a specific surface on chain B of CLL-1 in four of five experiments (Figures 6A,B). In other words, the top four binding poses (Figure 6A) demonstrate recurrent binding at the interface between chain A of CLL-1 and aptamer-2, suggesting a conserved interaction hotspot. A high-resolution close-up (Figure 6B) reveals specific molecular interactions, including hydrogen bonds and electrostatic contacts, stabilizing the aptamer-protein complex.

Structural analysis of the top-ranked pose (Figure 6C) highlights a deep binding groove in CLL-1, where the aptamer snugly fits. Surface mapping (Figure 6D) identifies key residues on chain B that form direct contacts with the aptamer, predominantly via electrostatic interactions and stacking with aromatic residues. Sites of interactions and residues are available in Supplementary Table S2.

These findings suggest that aptamer-2 exhibits a stable and specific binding mode to CLL-1, providing a structural framework for its potential application in molecular recognition strategies. Further validation through molecular dynamics simulations and experimental binding assays will refine our understanding of the aptamer’s affinity and specificity.

## 4 Discussion

Our study successfully identified the aptamer-2 sequence as a high-affinity, specific ligand for CLL-1, a myeloid surface protein implicated in acute myeloid leukemia (AML). Through iterative SELEX rounds, we observed progressive enrichment of CLL-1-binding aptamers, with aptamer-2 achieving a K_d_ of 1.55 × 10^−8^ M via SPR analysis, indicating nanomolar affinity comparable to antibodies and previous studies on aptamer targeting special protein (Zhou et al., 2025; Han et al., 2020). Flow cytometry further validated its specificity, with 94.3% of cells bound to CLL-1, surpassing other candidates (aptamer-1: 3.28%; aptamer-4: 2.84%). These results align with prior studies targeting AML biomarkers like Siglec-5, where aptamers demonstrated *K*_*d*_ values in the low nanomolar range (Yang et al., 2014). Our findings underscore the potential of aptamers as precision tools for early AML detection and targeted therapy (Han et al., 2020; Hori et al., 2018).

Our approach employed a magnetic bead-based SELEX protocol, combining rapid denaturation (95°C) and negative selection to eliminate non-specific binders. This mirrors methodologies used in cell-SELEX for AML biomarker discovery, such as the identification of Siglec-5-targeting aptamers (Yang et al., 2014). Notably, we incorporated eight iterative rounds of screening, a strategy shown to balance sequence diversity and enrichment efficiency (DeRosa et al., 2023). The secondary structure analysis revealed G-quadruplex motifs in aptamer-2, a feature associated with enhanced stability and target binding, as seen in PD-L1-targeting aptamers optimized via serum-assisted SELEX (Zhou et al., 2025). However, unlike advanced SELEX variants (e.g., serum-assisted or capture-SELEX), our method did not pre-adapt aptamers to physiological conditions, potentially limiting *in vivo* stability—a limitation addressed in recent work (Zhou et al., 2025).

While our study did not experimentally test commercial antibodies (e.g., anti-CLL-1 mAbs), aptamer-2’s affinity (K_d_ = 15.5 nM) aligns with reported antibody affinities (typically 1–10 nM) while offering aptamer-specific advantages: 30× faster synthesis (<72 h vs. weeks for antibodies), negligible batch variability, and thermal stability (denaturation-reversible) absent in biologics (Bauer et al., 2019; Jayasena, 1999). Critically, its 94.3% specificity exceeds Siglec-5-targeting aptamers (85% binding) and avoids the Fc-mediated off-target effects of antibodies (Yang et al., 2014).

CLL-1, a myeloid differentiation antigen, is a promising target for AML due to its overexpression on leukemic blasts. Our aptamer-2 outperformed controls in specificity assays, showing negligible binding to BSA—a critical advantage over non-specific interactions observed in earlier aptamer studies (Chen et al., 2025; Han et al., 2020). Comparatively, aptamers targeting PD-L1 in the mentioned study required serum-assisted SELEX to achieve stability in physiological environments (Zhou et al., 2025), whereas our aptamer-2 achieved sub-nanomolar affinity without such modifications. This contrasts with antibody-based systems, which often face challenges in scalability and stability despite high specificity (Altintas et al., 2025).

### 4.1 Limitations of this study

#### 4.1.1 Selection environment

Unlike serum-assisted SELEX (Zhou et al., 2025), our protocol used idealized buffer conditions, potentially underestimating aptamer degradation *in vivo*. However, a high affinity of aptamer-2 with CLL-1 may reduce its chance of degradation in *in vivo* situations. In addition, its 2D structure and predicted stability once again reduce its chance of degradation in a harsh medium or environment.

#### 4.1.2 Functional validation

While SPR and flow cytometry confirmed binding, *in vivo* efficacy and pharmacokinetics remain untested—a common gap in aptamer studies (Kovecses et al., 2024). This issue must be addressed by further investigations. In addition, in this study, direct comparative benchmarking was not performed among known mABs or other known aptamers against CLL-1. This issue should be considered for further studies.

#### 4.1.3 Target diversity

CLL-1 is one of many AML surface markers; broader screening (e.g., whole-cell-SELEX) could identify complementary targets, as demonstrated in Siglec-5 studies (Han et al., 2020).

#### 4.1.4 Clinical translation

Aptamer delivery to bone marrow niches, a challenge noted in nucleic acid therapeutics for AML (Kovecses et al., 2024), was not addressed here. Also, this study demonstrates the successful *in vitro* development and specificity of aptamer-2 for CLL-1 via flow cytometry and off-target assessment, a key limitation is the absence of functional validation in animal models or primary patient-derived samples. Although prior evidence supports consistent CLL-1 expression in AML, the current *in vitro* findings require future *in vivo* studies and evaluation using diverse patient samples to fully establish the aptamer’s therapeutic potential and clinical translatability.

These limitations should be addressed by researchers for further investigation and research studies.

### 4.2 Potential and perspective

#### 4.2.1 Diagnostic applications

The Aptamer-2 sequence could be integrated into biosensors for AML detection, leveraging advantages over antibodies (e.g., lower cost and tunability) (Sekhon et al., 2021). For instance, electrochemical aptasensors for PD-L1 have achieved picomolar sensitivity (Altintas et al., 2025), a benchmark our system could emulate.

#### 4.2.2 Therapeutic synergy

Combining aptamer-2 with differentiation agents (e.g., ATRA) or siRNA could enhance AML treatment, as proposed for nucleic acid therapeutics (Chu et al., 2006; Li et al., 2013).

## 5 Conclusion

In this study, we successfully identified and validated the aptamer-2 sequence as a highly specific and high-affinity candidate for targeting CLL-1 through a comprehensive and iterative SELEX process. Flow cytometry analysis across selection rounds demonstrated a progressive enrichment of aptamers, culminating in aptamer-2’s superior binding capability. The specificity of aptamer-2 was confirmed through rigorous validation, including negative controls and comparative fluorescence analysis, which highlighted its negligible non-specific binding and exceptional interaction with CLL-1. Surface plasmon resonance (SPR) measurements revealed an equilibrium dissociation constant (K_d_) of 1.55 × 10^−8^ M, underscoring its robust binding affinity.

These findings establish aptamer-2 as a promising molecular tool with significant potential for diagnostic and therapeutic applications targeting CLL-1. Its high specificity and binding strength position it as an ideal candidate for precise molecular recognition tasks, such as the development of diagnostic assays or targeted therapies for CLL-related conditions. This study not only confirms this aptamer’s potential but also provides a robust framework for the discovery and validation of aptamers for other clinically relevant targets. Future work may explore its functional applications in preclinical and clinical settings to further advance its utility in biomedical research and healthcare innovations.

## Data Availability

The original contributions presented in the study are publicly available. This data can be found here: https://pan.baidu.com/s/1j3wfeOyxN8fleYxPqKASwg?pwd=c58c.

## References

[B1] Altintas Z. Sehit E. Pan Y. Ma X. Yang Z. (2025). “Comparison of MIP-, Antibody- and aptamer-based biosensors for diagnostic technologies,” in Molecularly imprinted polymers: computational studies to advanced applications. Editor Altintas Z. (Cham: Springer International Publishing), 33–74.

[B2] Bakker A. B. van den Oudenrijn S. Bakker A. Q. Feller N. van Meijer M. Bia J. A. (2004). C-type lectin-like molecule-1: a novel myeloid cell surface marker associated with acute myeloid leukemia. Cancer Res. 64 (22), 8443–8450. 10.1158/0008-5472.can-04-1659 15548716

[B3] Bauer M. Strom M. Hammond D. S. Shigdar S. (2019). Anything you can do, I can do better: can aptamers replace antibodies in clinical diagnostic applications? Molecules 24 (23), 4377. 10.3390/molecules24234377 31801185 PMC6930532

[B4] Brown A. Brill J. Amini R. Nurmi C. Li Y. (2024). Development of better aptamers: structured library approaches, selection methods, and chemical modifications. Angew. Chem. Int. Ed. 63 (16), e202318665. 10.1002/anie.202318665 38253971

[B5] Chen Y. Li F. Zhang S. Liu F. Mao C. Li M. (2025). DNA framework-ensembled aptamers enhance fluid stability in circulating tumor cells capture for tumor treatment evaluation. Angew. Chem. 137, e202425252. 10.1002/anie.202425252 40040416

[B6] Chu T. C. Twu K. Y. Ellington A. D. Levy M. (2006). Aptamer mediated siRNA delivery. Nucleic acids Res. 34 (10), e73. 10.1093/nar/gkl388 16740739 PMC1474074

[B7] Daver N. Salhotra A. Brandwein J. M. Podoltsev N. A. Pollyea D. A. Jurcic J. G. (2021). A phase i dose-escalation study of DCLL9718S, an antibody-drug conjugate targeting C-type lectin-like molecule-1 (CLL-1) in patients with acute myeloid leukemia. Am. J. Hematol. 96 (5), E175–E179. 10.1002/ajh.26136 33617672 PMC8252033

[B8] DeRosa M. C. Lin A. Mallikaratchy P. McConnell E. M. McKeague M. Patel R. (2023). *In vitro* selection of aptamers and their applications. Nat. Rev. Methods Prim. 3 (1), 54. 10.1038/s43586-023-00238-7 PMC10647184 37969927

[B9] Didarian R. Ozbek H. K. Ozalp V. C. Erel O. Yildirim-Tirgil N. (2024). Enhanced SELEX platforms for aptamer selection with improved characteristics: a review. Mol. Biotechnol., 1–16. 10.1007/s12033-024-01256-w 39152308

[B10] Döhner H. Wei A. H. Löwenberg B. (2021). Towards precision medicine for AML. Nat. Rev. Clin. Oncol. 18 (9), 577–590. 10.1038/s41571-021-00509-w 34006997

[B11] Domsicova M. Korcekova J. Poturnayova A. Breier A. (2024). New insights into aptamers: an alternative to antibodies in the detection of molecular biomarkers. Int. J. Mol. Sci. 25 (13), 6833. 10.3390/ijms25136833 38999943 PMC11240909

[B12] Dunn M. R. Jimenez R. M. Chaput J. C. (2017). Analysis of aptamer discovery and technology. Nat. Rev. Chem. 1 (10), 0076. 10.1038/s41570-017-0076

[B13] Gómez-De León A. Demichelis-Gómez R. da Costa-Neto A. Gómez-Almaguer D. Rego E. M. (2023). Acute myeloid leukemia: challenges for diagnosis and treatment in Latin America. Hematology 28 (1), 2158015. 10.1080/16078454.2022.2158015 36607152

[B14] Han J. Gao L. Wang J. Wang J. (2020). Application and development of aptamer in cancer: from clinical diagnosis to cancer therapy. J. Cancer 11 (23), 6902–6915. 10.7150/jca.49532 33123281 PMC7592013

[B15] Hori S.-i. Herrera A. Rossi J. J. Zhou J. (2018). Current advances in aptamers for cancer diagnosis and therapy. Cancers 10 (1), 9. 10.3390/cancers10010009 29301363 PMC5789359

[B16] Jayasena S. D. (1999). Aptamers: an emerging class of molecules that rival antibodies in diagnostics. Clin. Chem. 45 (9), 1628–1650. 10.1093/clinchem/45.9.1628 10471678

[B17] Jiménez-García B. Roel-Touris J. Romero-Durana M. Vidal M. Jiménez-González D. Fernández-Recio J. (2018). LightDock: a new multi-scale approach to protein–protein docking. Bioinformatics 34 (1), 49–55. 10.1093/bioinformatics/btx555 28968719

[B18] Khwaja A. Bjorkholm M. Gale R. E. Levine R. L. Jordan C. T. Ehninger G. (2016). Acute myeloid leukaemia. Nat. Rev. Dis. Prim. 2 (1), 16010–16022. 10.1038/nrdp.2016.10 27159408

[B19] Kovecses O. Mercier F. E. McKeague M. (2024). Nucleic acid therapeutics as differentiation agents for myeloid leukemias. Leukemia 38 (7), 1441–1454. 10.1038/s41375-024-02191-0 38424137 PMC11216999

[B20] Kumar Kulabhusan P. Hussain B. Yüce M. (2020). Current perspectives on aptamers as diagnostic tools and therapeutic agents. Pharmaceutics 12 (7), 646. 10.3390/pharmaceutics12070646 32659966 PMC7407196

[B21] Li X. Zhao Q. Qiu L. (2013). Smart ligand: aptamer-mediated targeted delivery of chemotherapeutic drugs and siRNA for cancer therapy. J. Control. release 171 (2), 152–162. 10.1016/j.jconrel.2013.06.006 23777885

[B22] Mahmoudian F. Ahmari A. Shabani S. Sadeghi B. Fahimirad S. Fattahi F. (2024). Aptamers as an approach to targeted cancer therapy. Cancer cell Int. 24 (1), 108. 10.1186/s12935-024-03295-4 38493153 PMC10943855

[B23] Mori S. Nagae M. Yamasaki S. (2024). Crystal structure of the complex of CLEC12A and an antibody that interferes with binding of diverse ligands. Int. Immunol. 36 (6), 279–290. 10.1093/intimm/dxae006 38386511

[B24] Röthlisberger P. Hollenstein M. (2018). Aptamer chemistry. Adv. drug Deliv. Rev. 134, 3–21. 10.1016/j.addr.2018.04.007 29626546

[B25] Sacks N. C. Cyr P. L. Louie A. C. Liu Y. Chiarella M. T. Sharma A. (2018). Burden of acute myeloid leukemia among older, newly diagnosed patients: retrospective analysis of data from the 2010-2012 medicare limited data set. Clin. Ther. 40 (5), 692–703.e2. 10.1016/j.clinthera.2018.03.012 29673891

[B26] Sekhon S. S. Kaur P. Kim Y.-H. Sekhon S. S. (2021). 2D graphene oxide–aptamer conjugate materials for cancer diagnosis. npj 2D Mater. Appl. 5 (1), 21. 10.1038/s41699-021-00202-7

[B27] Shallis R. M. Wang R. Davidoff A. Ma X. Zeidan A. M. (2019). Epidemiology of acute myeloid leukemia: recent progress and enduring challenges. Blood Rev. 36, 70–87. 10.1016/j.blre.2019.04.005 31101526

[B28] Shin H. G. Yang H. R. Yoon A. Lee S. (2022). Bispecific antibody-based immune-cell engagers and their emerging therapeutic targets in cancer immunotherapy. Int. J. Mol. Sci. 23 (10), 5686. 10.3390/ijms23105686 35628495 PMC9146966

[B29] Tashiro H. Sauer T. Shum T. Parikh K. Mamonkin M. Omer B. (2017). Treatment of acute myeloid leukemia with T cells expressing chimeric antigen receptors directed to C-type lectin-like molecule 1. Mol. Ther. 25 (9), 2202–2213. 10.1016/j.ymthe.2017.05.024 28676343 PMC5589064

[B30] Wang J. Wang W. Chen H. Li W. Huang T. Zhang W. (2021). C-type lectin-like Molecule-1 as a biomarker for diagnosis and prognosis in acute myeloid leukemia: a preliminary study. BioMed Res. Int. 2021 (1), 6643948. 10.1155/2021/6643948 33778076 PMC7979301

[B31] Wang J. Zhao Y. Zhu C. Xiao Y. (2015). 3dRNAscore: a distance and torsion angle dependent evaluation function of 3D RNA structures. Nucleic acids Res. 43 (10), e63–e. 10.1093/nar/gkv141 25712091 PMC4446410

[B32] Yang M. Jiang G. Li W. Qiu K. Zhang M. Carter C. M. (2014). Developing aptamer probes for acute myelogenous leukemia detection and surface protein biomarker discovery. J. Hematol. Oncol. 7, 514. 10.1186/1756-8722-7-5 PMC3895837 24405684

[B33] Zhang Y. Xiong Y. Xiao Y. (2022). 3dDNA: a computational method of building DNA 3D structures. Molecules 27 (18), 5936. 10.3390/molecules27185936 36144680 PMC9503956

[B34] Zhou H. Li Y. Wu W. (2024). Aptamers: promising reagents in biomedicine application. Adv. Biol. 8 (6), 2300584. 10.1002/adbi.202300584 38488739

[B35] Zhou Y. Zhang H. Ding Y. Yu C. Li H. (2025). Serum assisted PD-L1 aptamer screening for improving its stability. Sci. Rep. 15 (1), 1848. 10.1038/s41598-025-85813-6 39805944 PMC11730316

[B36] Zhu C. Feng Z. Qin H. Chen L. Yan M. Li L. (2024). Recent progress of SELEX methods for screening nucleic acid aptamers. Talanta. 266, 124998. 10.1016/j.talanta.2023.124998 37527564

[B37] Zhuo Z. Yu Y. Wang M. Li J. Zhang Z. Liu J. (2017). Recent advances in SELEX technology and aptamer applications in biomedicine. Int. J. Mol. Sci. 18 (10), 2142. 10.3390/ijms18102142 29036890 PMC5666824

